# Microbial competition between *Escherichia coli* and *Candida albicans* reveals a soluble fungicidal factor

**DOI:** 10.15698/mic2018.05.631

**Published:** 2018-03-07

**Authors:** Damien J. Cabral, Swathi Penumutchu, Colby Norris, Jose Ruben Morones-Ramirez, Peter Belenky

**Affiliations:** 1 Department of Molecular Microbiology and Immunology, Division of Biology and Medicine, Brown University, Providence, RI, 02912, USA.; 2 Bryant University, Smithfield, RI, 02917 USA.; 3 Universidad Autónoma de Nuevo León, Facultad de Ciencias Químicas, Pedro de Alba, S/N, San Nicolás de los Garza, Nuevo León, México.; 4 Centro de Investigacion en Biotecnologia y Nanotecnologia, Universidad Autonoma de Nuevo Leon, Facultad de Ciencias Quimicas, Parque de Investigacion e Innovacion Tecnologica, Km. 10 autopista al Aeropuerto Internacional Mariano Escobedo, Apodaca, Nuevo Leon. 66629.

**Keywords:** microbial competition, Escherichia coli, Candida albicans, antifungal, magnesium

## Abstract

Localized and systemic fungal infections caused by *Candida albicans* can lead to significant mortality and morbidity. However, severe *C. albicans* infections are relatively rare, occurring mostly in the very young, the very old, and immunocompromised individuals. The fact that these infections are rare is interesting because as much as 80 percent of the population is asymptomatically colonized with *C. albicans. *It is thought that members of the human microbiota and the immune system work in concert to reduce *C. albicans *overgrowth through competition and modification of the growth environment. Here, we report that *Escherichia coli* (strain MG1655) outcompetes and kills *C. albicans *(strain SC5314)* in vitro.* We find that *E. coli* produces a soluble factor that kills *C. albicans *in a magnesium-dependent fashion such that depletion of available magnesium is essential for toxicity.

## INTRODUCTION

In order to combat the spread of antifungal resistance, we need to develop novel therapeutics to maintain our advantage over fungal infections. Microbial competition experiments have been an important tool for the discovery of novel antimicrobials since the work of Alexander Fleming, and the use of the "Waksman platform" to identify streptomycin and neomycin. These experiments can also provide important insight into the ways that microbial competition shape a healthy human microbial flora 1. The human flora consists of bacteria, fungi, viruses, and some parasitic eukaryotes 2. It is thought that this complex community and the interactions within it have coevolved to maintain relative homeostasis 2. In most cases, the human microbiota is a net benefit to the host, aiding in processes ranging from nutrition to protection from infection. Thus, the disruption of the healthy microbial community, termed dysbiosis, is associated with numerous negative health outcomes, including *Candida* overgrowth 3,4,5.

The nature of microbial interactions that maintain a healthy microbiota has been the subject of significant research since the turn of the twentieth century 6. We now understand that interactions within this community can be very complex, ranging from cooperation to predation 7,8. Significant work has also been done to identify interactions between commensal bacteria and fungal pathogens. For example, recent studies have indicated that *Staphylococcus aureus *mucosal infection is promoted via an interaction with invasive *C. albicans* hyphae 9. Similarly, *Candida* colonization of the respiratory tract may promote *Pseudomonas* *aeruginosa* infections in susceptible individuals 10,11. Interestingly *P. aeruginosa* has the opposite effect *in vitro*, interacting with fungal filaments to induce fungal death through the production of phenazine derivatives 12,13. *C. albicans *also forms mutually beneficial biofilms with a variety of pathogens and commensal bacteria, which increases pathogenicity while also providing bacteria protection from the host and antimicrobials 1,9,13. These polymicrobial communities may help commensal bacteria, but they can also promote fungal pathogenesis. For example, *E. coli* can contribute to bladder colonization and infection by *C. albicans* 14.

Not all *C. albicans-*bacteria interactions are mutually beneficial. In multiple examples, colonization with specific bacterial species has been shown to reduce *Candida* load and pathogenesis. Perhaps the most commonly used examples are vaginal and oral lactobacilli providing protection from vaginal and oral candidiasis, respectively 15. In fact, this is a somewhat controversial assessment, and the *in vitro* and *in vivo* data are inconclusive. Some studies indicate that pH reduction associated with lactic acid-producing bacteria (LAB) inhibits *C. albicans* growth *in vitro* 16,17, while other epidemiological studies do not show a significant correlation between LAB load and a reduction in fungal colonization or pathogenesis 1. Evidence from studies testing the probiotic impact of LAB—usually *Lactobacillus gasseri* and related species—is also mixed, with some studies showing robust protection from candidiasis and others showing no improvement over control or placebo 4,15,18,19.

In addition to the potential anti-*Candida* activity of lactobacilli, there is some evidence that enteric bacteria such as *E. coli* can be antifungal. The *E. coli* probiotic strain Nissle 1917 (serovar O6:K5:H1) provides protection from numerous pathogens *in vitro, *including *C. albicans *20. It is thought that this protection results from the immunomodulatory activity of Nissle, although this assumption has not been directly tested 21,22,23. In addition to Nissle, several other *E. coli* strains have been found to inhibit *C. albicans* 24. Hummel *et al*. performed both *in vitro* and *in vivo* studies, finding that naturally occurring *E. coli* strains can not only inhibit fungal colonization, but also induce a zone of inhibition on a *Candida* lawn. This result indicates that the anti*-Candida* activity of *E. coli* may utilize a soluble factor in addition to immunomodulation. Other studies have also found that *E. coli* and its lipopolysaccharide can modulate fungal growth and biofilms *in vitro *25,26*. *Together, this work indicates that *E. coli* could inhibit and possibly kill *C. albicans* during a polymicrobial interaction. To decipher this interaction, we chose to co-culture *E. coli* (strain MG1655) and *C. albicans *(strain SC5314) *in vitro*. We found that *E. coli* rapidly kills *C. albicans *via a soluble factor in a manner that is inversely dependent on the availability of magnesium.

## RESULTS AND DISCUSSION

**Figure 1:**
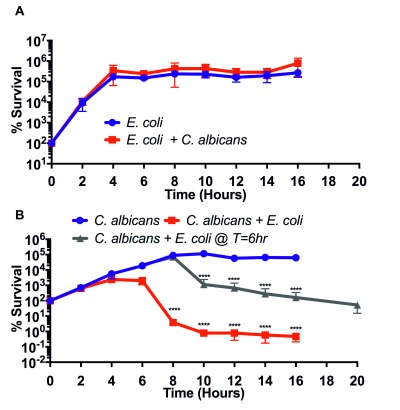
FIGURE 1: *E. coli* kills *C. albicans* in culture. **(A)** Percent survival of *E. coli* cultured alone or in co-culture with *C. albicans.* **(B)** Percent survival of *C. albicans* cultured alone or in co-culture with *E. coli *added at T=0 hours or at T= six hours*.* Data shown reflect mean ± SD of n = 3. Statistical significance was calculated using a two-way ANOVA in Prism 7.0. For each time point, both *C. albicans *+ *E. coli* media types were compared to the *C. albicans* media control. P-values were subsequently corrected for multiple comparisons using the Bonferroni method. **** denotes an adjusted p-value of less than 0.0001. No time points were found to be statistically significant in Figure 1A.

To define the interaction between *E. coli* and *C. albicans* *in vitro,* we cultured both organisms as monocultures and co-culture in YPD medium at 37(C with vigorous shaking. Serial dilutions of the cultures were plated on solid YPD supplemented with ampicillin to quantitate *C. albicans *and solid LB supplemented with amphotericin B to quantitate *E. coli*. We found that *E. coli *grew equally well in the presence and absence of *C. albicans* (Figure 1A). Conversely, *C. albicans *growth was impacted by *E. coli *co-culture when compared to *C. albicans* grown alone, showing growth inhibition by six hours (Figure 1B). Interestingly, the number of viable *Candida* cells began to decrease after six hours of culture and was eventually reduced 5000-fold by 16 hours when compared to the six-hour time point. This result indicates that *E. coli* is not only inhibitory of *C. albicans* but also robustly fungicidal. To determine whether fungal cell numbers or growth phase was important for fungicidal activity, we delayed the addition of *E. coli *until 6 hours after the initiation of *Candida* culture - about the time *C. albicans* reaches maximum density. We found that the bacteria had a robust fungicidal effect even when the *E. coli* was added to fully dense fungal culture, reducing fungal counts by approximately 1500-fold from the maximum density (Figure 1B). This indicates that *E. coli* can induce fungal death even in dense culture and demonstrates that *E. coli *has robust fungicidal activity.

**Figure 2:**
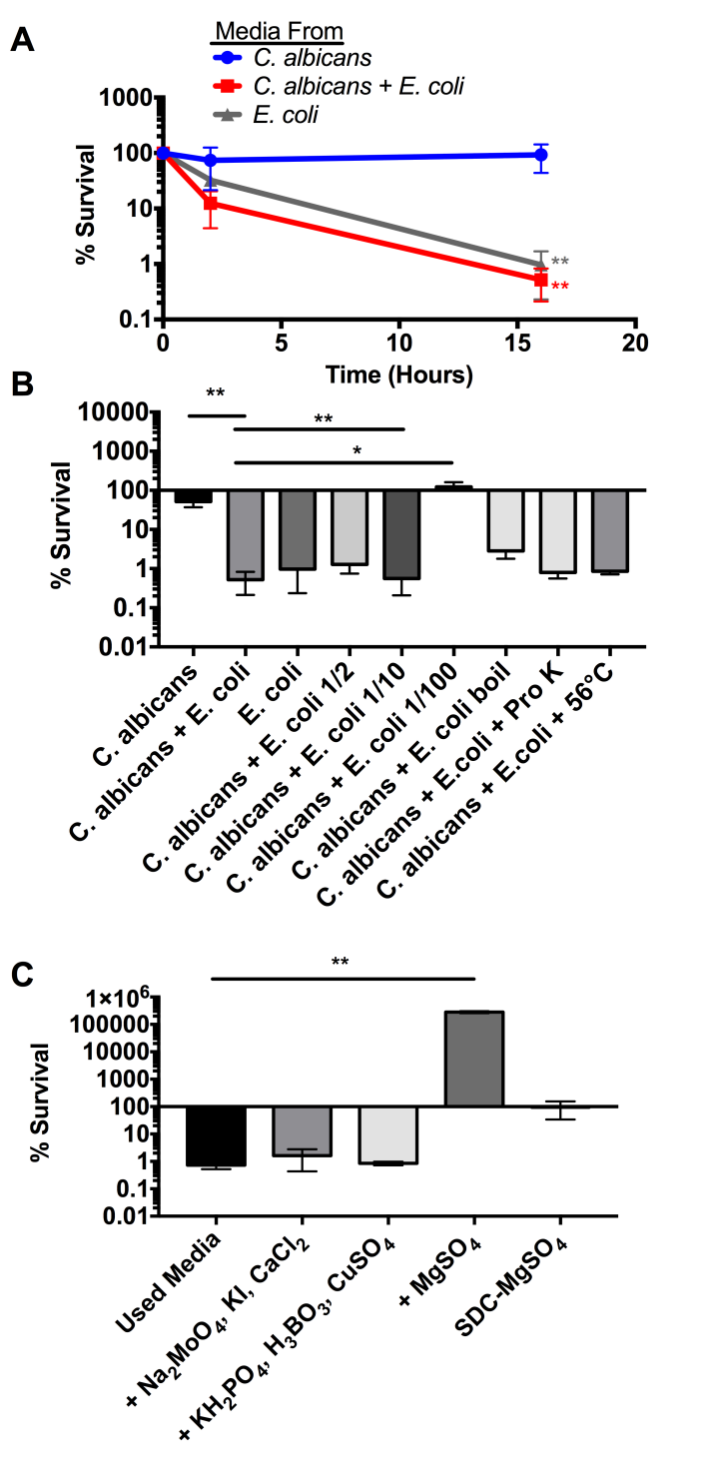
FIGURE 2*: E. coli* produces a soluble fungicidal molecule. **(A)** Percent survival of *C. albicans* cultured in the indicated conditioned media collected after 6 hours of co-culture*.* Statistical significance was calculated using an unpaired two-tailed t-test in which the *E. coli* and the *C. albicans + E. coli* medias were compared to the *C. albicans* media control. **(B)** Percent survival of *C. albicans* cultured in the indicated conditioned media for 16 hours. Statistical significance was calculated using a one-way ANOVA in Prism 7.0. P-values were subsequently corrected for multiple comparisons using the Bonferroni method. All conditions were compared to the *C. albicans* media control. **(C)** Percent survival of *C. albicans* cultured in *E. coli* + *C. albicans* conditioned media for 16 hours. Compounds were added at the concentrations consistent with standard YNB [Boric acid (0.5 mg/L), Copper (II) sulfate pentahydrate (0.04 mg/L) Sodium molybdate (0.2 mg/L), Potassium iodide (0.1 mg/L), Calcium chloride dihydrate (100 mg/L), Potassium phosphate monobasic anhydrous (1000 mg/L) and Magnesium sulfate anhydrous (500 mg/L)]. Statistical significance was calculated using a one-way ANOVA in Prism 7.0 to compare all conditions to the used media control. P-values were subsequently corrected for multiple comparisons using the Bonferroni method. Statistical significance is shown (*p > 0.05; **p > 0.01 ***p > 0.001).

Our data indicate that *E. coli* can induce conditions that lead to fungal death during *in vitro* co-culture with *C. albicans*. This phenotype can result from close interaction between bacterial cells and fungal cells, as is seen in the case of *Candida *and *P. aeruginosa* 12, or from the production of a fungicidal compound. To test whether *E. coli* produces a toxic soluble factor, we collected conditioned media at six hours from *C. albicans* and *E. coli* monocultures and from the co-culture. The respective conditioned media were filtered and buffered with PBS (pH 7.4) prior to the addition of fresh log-phase *C. albicans *cells. We found that over a 16-hour experiment, cells grown in *C. albicans* conditioned media did not display a reduction in colony forming units (CFU). On the other hand, *E. coli *conditioned media and media from the co-culture induced a 100-fold drop in viable CFUs (Figure 2A). We also found that media from the co-culture was somewhat more toxic than media from *E. coli* alone, although the difference was not consistently statistically significant. These results indicate that *E. coli* secretes a powerful fungicidal element and that its production may not be significantly stimulated by the presence of *C. albicans.*

To further explore the impact of conditioned media on fungal death, we conducted additional endpoint experiments that quantified fungal viability after 16 hours (Figure 2B). If fungal toxicity in conditioned media resulted from the presence of a soluble factor, we would anticipate that the toxic activity would dissipate with dilution. We found that diluting the co-culture conditioned media with PBS did not significantly impact toxicity at a 1:2 dilution and at a 1:10 dilution (Figure 2B). However, the 1:100 dilution completely abolished fungicidal activity, indicating that the *E. coli*-produced factor behaves as a dilutable molecule. Next, we sought to test the thermal stability of the fungicidal activity by boiling the co-culture conditioned media for ten minutes prior to testing fungicidal activity. We found that boiling reduces fungicidal activity by an order of magnitude but did not completely abolish it, indicating that the fungicidal molecule is relatively heat stable. Other studies have indicated that bacteria can produce bacteriocins like fungicidal peptides 27,28. We suspect that *E. coli* could be producing a similar fungicidal peptide. We exposed co-culture conditioned media to proteinase K (Pro K) digestion for 2 hours at 56°C. This treatment did not reduce antifungal activity, indicating that the secreted molecule is insensitive to Pro K under the tested conditions (Figure 2 B). However, this is not a conclusive indication that the antifungal molecule is not a peptide. It is possible that Pro K activity was inhibited by conditioned media components or that the peptide sequence/structure is not cleavable by Pro K.

During this work, we noticed reduced fungicidal activity when the co-culture was conducted in different batches of YPD powder purchased from Fisher Scientific. While this result was rare, it indicated that media content might play a role in the competitive interaction and fungicidal toxicity of the produced factor. YPD is not a defined media, thus some variability is possible from batch to batch. However, yeast nitrogen base (YNB), typically used to make synthetic dextrose complete (SDC) media, is defined, containing set concentrations of vitamins, metals, and trace elements that are essential for fungal growth. Since metals and trace elements are most likely to be limiting in growth media, we chose to focus on these molecules. To test the impact of metals and trace elements on toxicity, we added either single compounds or mixtures of compounds at concentrations found in YNB to our conditioned co-culture media. We found that the addition of magnesium alone was sufficient to disrupt all fungicidal activity and actually restore fungal growth in previously toxic conditioned media. This result may seem to indicate that magnesium limitation is the cause of the toxicity. However, this is not the case because Figure 1B demonstrates that the toxicity is dilutable and heat labile. If magnesium limitation were the sole cause of toxicity, we would anticipate that dilution of conditioned media would enhance fungicidal activity rather than abolish it. Additionally, when *C. albicans *was grown in media lacking magnesium (SDC - Mg), we observed growth arrest but not toxicity (Figure 2C). While these results do not indicate that magnesium limitation is the cause of toxicity, they do indicate that efficient killing requires magnesium limitation.

**Figure 3:**
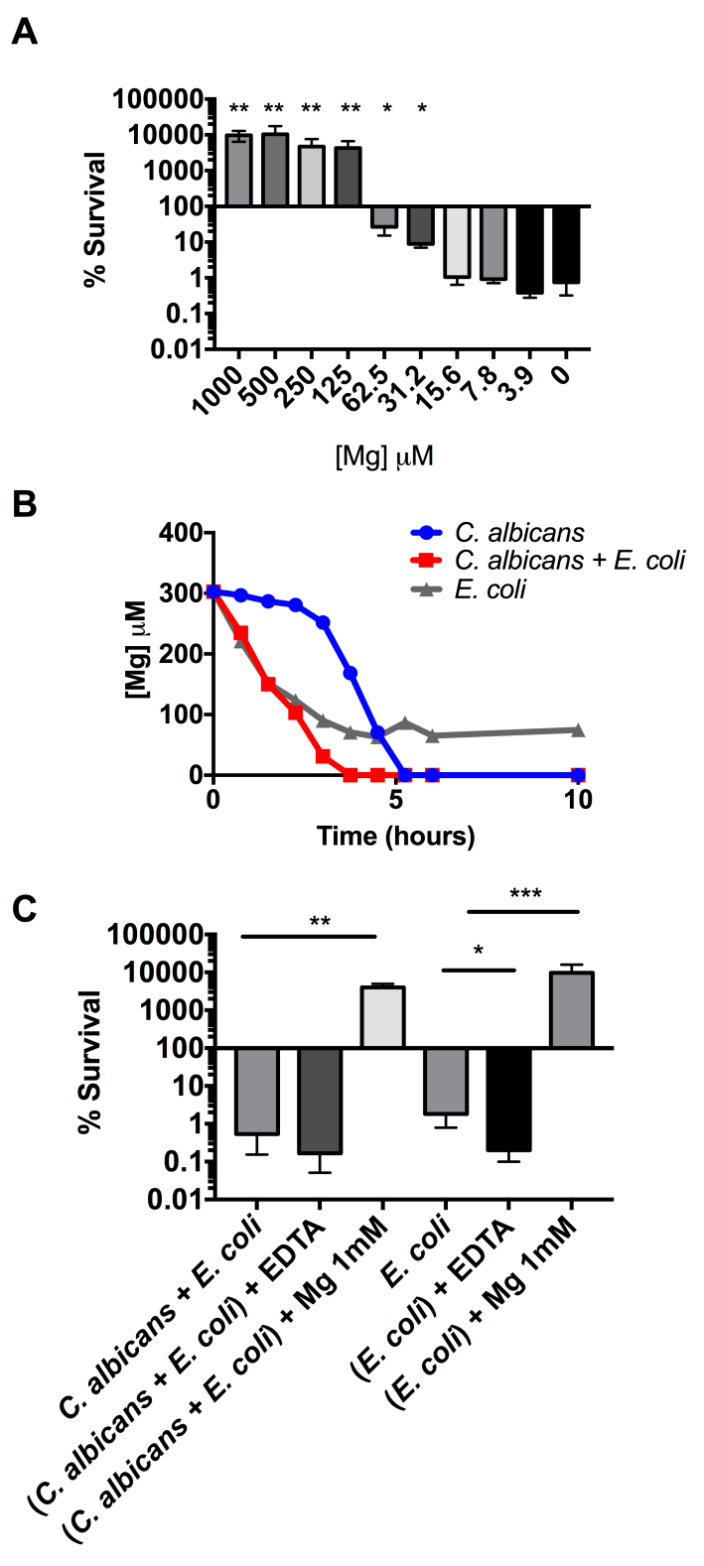
FIGURE 3: *E. coli* toxicity depends on magnesium limitation. **(A)** Percent survival of *C. albicans* cultured in *E. coli* + *C. albicans* conditioned media with the indicated concentrations of added MgSO_4_. To calculate statistical significance, percent survivals were first log_10_ normalized to stabilize variances. Paired, two tailed t-tests were performed to compare every condition to the negative control (0 (M). **(B)** Quantified magnesium concentration in condition media. **(C)** Percent survival of *C. albicans* cultured in the indicated conditioned media for 16 hours. EDTA was added at 500 μM final concentration. Data shown reflect mean ± SD of n = 3. Statistical significance was calculated using a paired two-tailed t-test (*p > 0.05; **p > 0.01 ***p > 0.001).

To further explore the impact of magnesium on toxicity, we titrated varying amounts of MgSO_4_ into co-culture conditioned media. We found that the addition of magnesium begins to reduce toxicity at concentrations above 15 (M and promotes growth after 125 (M (Figure 3A). This indicates that magnesium limitation may be the cause of *C. albicans *growth limitation in YPD. To quantify magnesium levels in conditioned media at multiple time points, we utilized a colorimetric magnesium assay kit (MAK026 SIGMA). We found that in a YPD *C. albicans*-*E. coli *co-culture, magnesium levels are completely depleted by four hours (Figure 3B) - a time that coincides with the first detectable toxicity during co-culture (Figure 1B). The depletion of magnesium by *C. albicans* alone displayed different kinetics but was also complete by five hours. This result supports our assessment that magnesium depletion alone is not sufficient to induce toxicity because we found that conditioned media collected from a *C. albicans* monoculture was not toxic despite the complete depletion of magnesium by six hours (Figure 3B). Finally, we found that magnesium levels drop rapidly but do not fall below 60 (M in the *E. coli *monoculture. This may explain the slightly reduced toxicity of *E. coli* conditioned media when compared to the co-culture.

Next, we tested the impact of ethylenediaminetetraacetic acid (EDTA), a magnesium chelator, on the toxicity of conditioned media. We found that the addition of EDTA did not have a statistically significant impact on the toxicity of the co-culture conditioned media. However, it did have a significant impact on the toxicity of *E. coli* monoculture media, restoring it to the same toxicity as the co-culture media. Combined with results from Figure 3B this observation may indicate that chelating the remaining magnesium in the *E. coli* media promoted toxicity, further supporting the role of magnesium depletion in *E. coli*-mediated killing of *C*. *albicans*. However, because EDTA can also chelate other divalent ions, such as calcium, we must also consider the possibility that these other ions may play a role.

## CONCLUSION

In this work, we found that *E. coli* kills *C. albicans* when co-cultured *in vitro*. We assert that this activity results from a soluble factor produced by *E. coli* in a manner that is independent of the presence of fungal cells. We also found that magnesium limitation is required for the observed toxicity. While we hope that this work can lead to the identification of novel antifungal therapeutics in the future, we are also cognizant that the requirement of magnesium limitation may somewhat limit the therapeutic use or clinical relevance of our discovery. The total magnesium in human serum ranges from between 1000 and 500 (M 29. This is significantly higher than the maximum concentration under which we see toxicity *in vitro*. This indicates that if the toxic *E. coli*-*C. albicans* interactions were to occur *in vivo*, they would need to happen in microenvironments within the host where magnesium concentrations are naturally lower or depleted by fungal or bacterial growth; however, we envision that local conditions in the host are sufficient to deplete magnesium, promote interactions between *E. coli *and *C. albicans*, and induce the observed fungicidal activity.

We view this work as a critical first step in defining the identified competitive interaction between *Candida *and *E. coli*. The next steps should involve the fractionation of conditioned media for the identification of the active compound and the screening of *E. coli* knockouts to identify genes required for its production. Perhaps the most important and interesting observation presented here is the role of magnesium limitation in toxicity. Thus, explaining this observation could be the most interesting next step. We hypothesize that three potential factors could explain this observation. First, the toxic compound produced by *E. coli* may only be active on non-growing fungal cells and thus magnesium limitation promotes toxicity by blocking fungal growth. Second, the compound may require active import that is induced by magnesium restriction. Finally, the compound may be inhibited by magnesium binding. By identifying how magnesium regulates toxicity, we may find methodologies that maintain toxicity under physiological conditions.

Based on magnesium ion sensitivity and heat instability, we suspect that the detected antifungal activity is likely caused by a peptide. Bacteria produce a wide array of antimicrobial bacteriocin peptides and bacteriocin-like extracellular metabolites to control surrounding microbial populations 30,31. Some of these bacteriocins can also display significant antifungal activity 27,28,32. Like other bacteria, *E. coli* also produces multiple bacteriocins, including colicins and microcins 33,34. While these are mostly antibacterial in nature, it is possible that bacteriocins produced by *E. coil* under our experimental conditions have antifungal properties. Most intriguingly, some studies indicate that bacteriocin toxicity is impacted by divalent cations such as magnesium. For example, the antimicrobial activity of a bacteriocin from *Streptococcus sanguinis* is accentuated with decreasing external concentrations of Ca^2+^ 30. In other cases, antimicrobial properties are increased when Ca^2+^, Fe^+3^ and Mg^+2^ concentrations are increased 35,36,37. Thus, the modulation of bacteriocin activity by divalent cation concentrations presents a possible connection to our observed magnesium results.

## MATERIALS AND METHODS

Co-culture experiments were conducted in YPD incubated at 300 rpm and 37°C. YPD liquid medium was sterilized by filtration rather than autoclaving to preserve nutritional content. Overnight fungal cultures of *C. albicans *(strain SC5314) were diluted to an OD_600_ of 0.07 and allowed to grow to an OD_600_ of 0.2. Overnight cultures of *E. coli* (strain MG1655) were grown in LB and were subsequently diluted 1:200 into fresh YPD and allowed to grow to an OD_600_ of 0.2. When both fungal and bacterial cultures reached 0.2, they were centrifuged and re-suspended in fresh YPD. Final culture experiments were started in 250 mL flasks (25 mL of culture) with *C. albicans *diluted to an OD_600_ of 0.1 and *E. coli* diluted to 0.001. Over the next 16 to 20 hours, CFUs were measured by plating eight serial dilutions onto YPD agar plates with 100μg/mL of ampicillin (to detect *C. albicans)* and on LB with 100 μg/mL of amphotericin B (to detect *E. coli)*. CFU/mL was converted to percent survival for analysis.

Conditioned media was made by filtering growth media through a 0.2-micron filter and by adding PBS (pH 7.4) to a final concentration of 0.1X. The final solution was stored for up to two weeks at 4(C. Conditioned media experiments were initiated by diluting PBS-washed log-phase *C. albicans* into 0.5 mL of conditioned media to an OD_600_ of 0.1. Kill curves were conducted in 24-well plates incubated at 300 rpm and 37°C. CFUs were again measured by plating eight serial dilutions onto YPD agar plates.

Pro K treatment was conducted by incubating conditioned media buffered with 0.1X PBS with 100 µg/mL of Pro K (NEB) for two hours at 56°C. Magnesium was quantitated in conditioned media using a colorimetric magnesium assay kit (MAK026 SIGMA) according to manufacturer’s instructions.
